# Effect of Fasting on the Spexin System in Broiler Chickens

**DOI:** 10.3390/ani11020518

**Published:** 2021-02-17

**Authors:** Paweł A. Kołodziejski, Ewa Pruszyńska-Oszmałek, Marcin Hejdysz, Maciej Sassek, Natalia Leciejewska, Kamil Ziarniak, Jakub Bień, Piotr Ślósarz, Marta Kubiś, Sebastian Kaczmarek

**Affiliations:** 1 Department of Animal Physiology, Biochemistry and Biostructure, Poznan University of Life Sciences, 60-637 Poznan, Poland; ewa.pruszynska@up.poznan.pl (E.P.-O.); maciej.sassek@up.poznan.pl (M.S.); natalia.leciejewska@up.poznan.pl (N.L.); jakub.bien@up.poznan.pl (J.B.); 2 Department of Animal Breeding and Product Quality Assessment, Poznan University of Life Sciences, Sloneczna 1, 62-002 Zlotniki, Poland; marcin.hejdysz@up.poznan.pl (M.H.); piotr.slosarz@up.poznan.pl (P.Ś.); 3 Laboratory of Neurobiology, Department of Zoology, Faculty of Veterinary Medicine and Animal Science, Poznan University of Life Sciences, 60-625 Poznan, Poland; kamil.ziarniak@up.poznan.pl; 4 Department of Preclinical Sciences and Infectious Diseases, Faculty of Veterinary Medicine and Animal Science, Poznan University of Life Sciences, 60-637 Poznan, Poland; 5 Department of Animal Nutrition, Poznan University of Life Sciences, 60-637 Poznan, Poland; marta.kubis@up.poznan.pl (M.K.); sebastian.kaczmarek@up.poznan.pl (S.K.)

**Keywords:** spexin, broiler chicken, fasting

## Abstract

**Simple Summary:**

The regulation of physiological processes by biologically active substances such as peptides, proteins, or hormones is very important in the context of both the development of the basic sciences and their subsequent use in improving animal husbandry. One such substance is spexin (SPX), a recently discovered, very conservative peptide that has been shown in mammalian studies to be able to regulate food intake, as well as carbohydrate–lipid metabolism. Because there is no information on the role of SPX in the metabolism of birds in the literature, we first decided to determine whether the expression of the gene encoding this peptide is present in the various tissues of birds. A further object of the study was to determine whether the concentration of SPX in serum blood changes during the disturbance of the carbohydrate metabolism caused by starvation and whether these changes correlate with other metabolic parameters. These studies will help us fully understand the role of SPX in bird physiology, and this research should be further extended.

**Abstract:**

Spexin (SPX) is a highly conservative peptide hormone containing 14 amino acids and was discovered in 2007 by bioinformatics methods. However, nothing is yet known about its role in the metabolism of birds, including broilers. The aim of this study was to investigate the effect of short-term fasting (2, 4, and 8 h) on the concentration of SPX in blood serum and the expression levels of the genes encoding this peptide (*SPX1*) and its receptors, GALR2 and GALR3, in the tissues involved in carbohydrate and lipid metabolism (muscles, adipose tissue, and liver). We also analyzed the mRNA expression of these genes in various chicken tissues. Moreover, we studied the correlation between the serum level of SPX and other metabolic parameters (insulin, glucagon, glucose, triglycerides, and cholesterol). Using RT-qPCR, we found that *SPX1, GALR2,* and *GALR3* are expressed in all investigated tissues in broiler chicken. Moreover, using a commercially available radio-immunoassay, we noted an increase of the SPX level in blood serum after 4 and 8 h of fasting compared to nonfasted animals (*p* < 0.05). This increase was positively correlated with glucagon concentration (r = 0.341; *p* < 0.05) and negatively with glucose concentration (r = −0.484; *p* < 0.01). Additionally, we discovered that in the short term, food deprivation leads to the expression regulation of *SPX1*, *GALR2,* and *GLAR3* in tissues associated with metabolism of carbohydrates and lipids. The obtained results indicate that SPX is involved in the regulation of metabolism in broiler chickens.

## 1. Introduction

In recent years, increasingly more peptides, proteins, and biologically active substances have been discovered thanks to the use of new techniques, such as bioinformatics tools. One of these peptides is spexin (SPX), which was discovered in 2007 using the hidden Markov model by Mirabeau et al. [1]. This peptide is highly conserved, with a length of 14 amino acids. Analysis showed that the amino acid sequence of SPX is identical in humans, rats, mice, and chickens [2]. The first reports on the role and origin of this peptide showed it to belong to the same peptide family as kisspeptin and galanin [3]. The biological activity of SPX is regulated via two isoforms of the galanin receptor: GALR2 and GALR3 [3]. The most important functions of spexin that have been described in the literature so far include the inhibition of food intake, the regulation of body weight and fat tissue, and liver metabolism, as well as reproductive functions [4,5,6,7,8]. Moreover, some research showed that SPX is able to enhance peristaltic movements [9] and regulate insulin and glucose metabolism in mammals and fishes [10,11,12].

Despite the growing knowledge of SPX in many animal species, there is no data on its expression and role in the metabolism of birds. Therefore, we decided to investigate the expression of this peptide (*SPX1*, a gene-variant-encoding peptide with a similar amino acid sequence to that in humans and mice; *GALR2* and *GALR3*) at the mRNA level in various tissues of broilers. Moreover, we examined the effect of short-term starvation on the level of SPX in the blood, as well as its expression of *SPX1* in the tissues involved in glucose metabolism, including adipose tissue, liver, and muscle tissue.

## 2. Materials and Methods

### 2.1. Birds and Diets

Chicks were obtained from a local commercial hatchery (DanHach Poland, Wolsztyn, Poland). ROSS 308 birds (*n* = 36) were kept on a wood-shaving litter in pens (three birds per pen) with an area of 2 m^2^. During the first seven days, the birds were exposed to light for 24 h/day, followed by 18 h light/6 h darkness (28 days). In line with the AVIAGEN 2014 guidelines, the temperature was maintained at 32 °C during the first week and was gradually decreased to approximately 23 °C by the end of the experiment. Birds were fed two types of diets: a starter diet from 1 to 14 days of life and a grower diet from 15 to 35 days of life. The diets (Table 1) were formulated based on the analyzed chemical composition of maize and the soybean meal and the nutritive value of feedstuffs published by Smulikowska and Rutkowski [13]. Nutrients met or exceeded the breeder recommendations for broiler chickens [14].

Diets were pelleted using a Scorpion pellet press (BMG Pelleting Experts, Gdańsk, Poland). The pelleting conditions were monitored and maintained at a constant ampere draw of the load meter for the mill motor to ensure consistency in pelleting conditions. The die size was 3 mm, and the rate was around 100 kg/h. For the starter diet, pellets were additionally crumbled. All experiments were carried out in accordance with Polish law and regulations regarding experiments with animals.

### 2.2. Sampling

Chickens (*n* = 32) aged 35 days were divided into four equal groups, a control group and three experimental groups, which were fasted for 2, 4, and 8 h. After this time, the birds were sacrificed by electrical stunning. The blood and tissues (pancreas, liver, lung, kidney, spleen, thigh muscle, breast muscle, cecum, ileum, duodenum, proventriculus, gizzard, heart, visceral fat, and spinal cord) were collected for further analysis. Tissues were placed immediately after harvesting in liquid nitrogen and stored at −80 °C. The blood was centrifuged (3500 rpm, 15 min, 4 °C), and the obtained serum was stored at −20 °C.

### 2.3. Chemical Analyses

The representative samples of feed were ground and passed through a sieve with a mesh size of 0.5 mm. The diets were then analyzed in duplicate for dry matter (DM), crude protein (CP), crude fat (CF), Ca, and P, using the methods of AOAC (2005) [15]. Nitrogen content was analyzed using a Kjel Foss Automatic 16,210 analyzer (A/S N. Foss Electric, Hillerød, Denmark), and CF was determined using a Soxtec System HT 1043 Extraction Unit (Foss Tecator, Denmark).

### 2.4. Biochemical Profile

The biochemical profile was measured using commercially available kits. The determinations were made in accordance with the instructions attached by the test manufacturer with modifications performed by adapting the test to the method using 96-well microplates. The optical density of the samples was measured using a Synergy 2 microplate reader (Biotek, Winooski, VT, USA). The levels of glucose (Cat. No.: G7519), triglycerides (TG; Cat. No.: T7531), total cholesterol (TCh; Cat. No.: C7510), albumin (Cat. No.: A7502), and total protein (Cat. No.: T7528) were measured using kits from Pointe Scientific (Lincoln Park, MI, USA).

### 2.5. Hormonal Profile

The concentration of SPX was measured using SPEXIN RIA Kit (cat. No RK-023-81; sensitivity: 20 pg/mL; standard range: 5–640 pg/mL; crossreactivity: 0%; Phoenix Pharmaceuticals, Burlingame, CA, USA). The radioactivity of the samples was detected using a Wizard 2 Gamma Counter (Perkin Elmer, Norwalk, CT, USA). All samples were determined in duplicate. Insulin and glucagon concentrations were measured using insulin and glucagon radio-immunoassays (RIA) kit (cat. No. RI-13K and GL-32K, respectively, Merck Millipore, Burlington, MA, USA) [16,17].

### 2.6. Real-Time PCR

The total RNA from different tissues was isolated using an Extrazol Reagent (Cat. No.: EM30-200; DNA Gdansk, Gdansk, Poland). Quality and quantity of RNA of isolated RNA were determined using a UV-VIS Spectrophotometer: NanoPhotometer^®^ NP80 (Implen GmbH, München, Germany). Additionally, the integrity of RNA was determined by electrophoresis in 1% agarose gel. cDNA was synthetized using 1 ug of total RNA with a high-capacity cDNA reverse-transcription kit (Cat. No.: 4368813; Applied Biosystems, Forster City, CA, USA). DNA contamination of samples was excluded by performing RT-PCRs in parallel without added RT and detected no signals. For primer design, Primer Blast software was used (Primers sequences are presented in Table 2). Real-time PCR was performed using QuantStudio 12 K Flex™ real-time PCR (Life Technologies, Grand Island, NY, USA) and 5 x HOT FIREPol^®^ EvaGreen^®^ qPCR Mix Plus (ROX) (Cat. No.: 08-24-00008; Solis BioDyne, Tartu, Estonia). Amplification involved one cycle at 95 °C for 12 min for initial denaturation and then 45 cycles consisting of denaturation (95 °C for 15 s), annealing (61.5 °C for 30 s), and elongation (72 °C for 20 s). The fluorescent product detection was performed at the last step of each cycle. Specificity of amplification was determined by analyses of product melting after each reaction (0.1 °C/s increment from 65 to 95 °C, with fluorescence collection at 0.1 °C intervals). Relative gene expression was evaluated by Delta Delta CT (ΔΔCT) with β-actin as a reference gene.

### 2.7. Statistical Analysis

Results are presented as the arithmetic mean ± SEM. The significance of differences was determined using a one-way analysis of variance (ANOVA) with a Dunnett post hoc test compared to the control group (time 0) * *p* < 0.05 and ** *p* < 0.01. Relationships between the SPX concentration and other parameters of blood were analyzed using Pearson’s correlation model and linear regression. Correlation coefficient values below 0.3 were considered weak, those between 0.3 and 0.5 as mild, those from 0.5 to 0.7 as moderate, and those 0.7 or greater as high correlation.

## 3. Results

### 3.1. Tissue Expression of SPX1, GALR2, and GALR3

**Figure 1:**
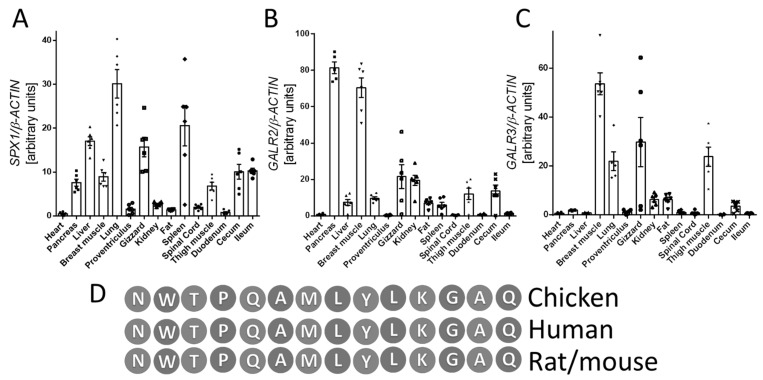
mRNA expression of SPX1 (**A**), GALR2 (**B**), and GALR3 (**C**) genes in various chicken tissues. Comparison of SPX amino acid sequences in chickens, humans, mice, and rats (**D**). Results (graph: **A**–**C**) are shown as the means ± SEMfor each value (*n* = 6). The dots and symbols represent the individual values obtained.

First, we investigated the expression profiles of *SPX1*, *GALR2*, and *GALR3* in various tissues obtained from broiler chickens (heart, pancreas, liver, breast muscle, lung, glandular stomach, muscular stomach, kidney, visceral fat, spleen, spinal cord, thigh muscle, duodenum, cecum, and ileum). We observed mRNA *SPX1*, *GALR2*, and *GALR3* expression in all investigated types of tissues. We found the highest expression of *SPX1* in pancreas, liver, breast muscle, lung, muscular stomach, spleen, thigh muscle, cecum, and ileum. The expression level of *GALR2* and *GALR3* mRNA was more varied, as shown in Figure 1.

### 3.2. Effect of Fasting on the SPX Concentration in Blood Serum.

**Figure 2:**
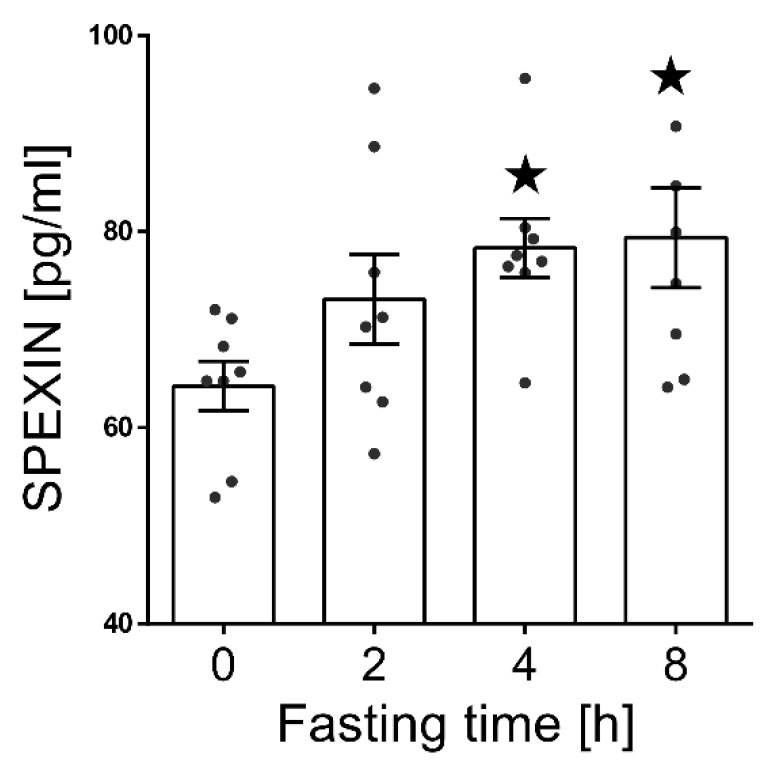
Serum SPX concentration changes during short-term fasting. Results are shown as the means ± SEM for each value (*n* = 8). The dots represent the individual values obtained. Statistically significant changes are marked as asterisk symbol * *p* < 0.05 compared to time 0.

We observed a statistically significant increase of the serum SPX level (*p* < 0.05) after 4 and 8 h of fasting compared to nonfasting animals (Figure 2).

### 3.3. Effect of Fasting on Basic Metabolic and Hormonal Profile.

We also investigated the effect of fasting on the concentration of glucose, TG, TCh, albumin, and total protein, as well as insulin and glucagon levels, in the blood serum. We found a lower concentration of glucose (Table 3; *p* < 0.01) and TG (Table 3; *p* < 0.05) after 4 and 8 h of fasting the birds. We also observed a decrease in the insulin level at the last measuring point—8 h (Table 3; *p* < 0.05). At the same time, we noted an increase in glucagon concentration after 4 (Table 3; *p* < 0.05) and 8 h (Table 3; *p* < 0.01) and NEFA (Non-esterified fatty acids) concentration after 4 (Table 3; *p* < 0.05) and 8 h (Table 3; *p* < 0.01) of food depravation.

### 3.4. Correlation of SPX Concentration with Others Metabolic Parameters

**Figure 3:**
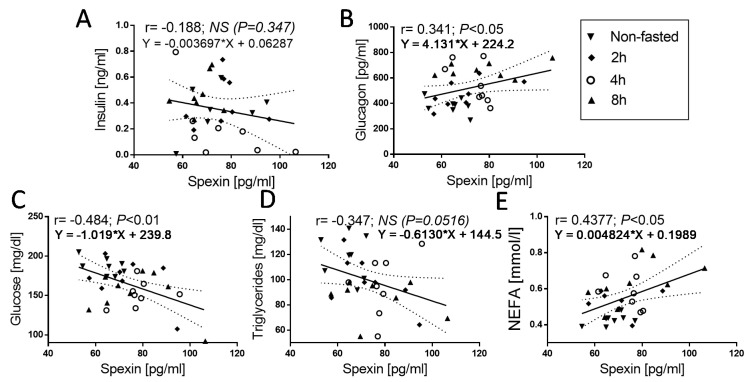
Correlations between circulating SPX concentration and insulin (**A**), glucagon (**B**), glucose (**C**), triglycerides (**D**), and NEFA (**E**) in nonfasted and 2, 4, and 8 h fasted chickens. The values for r and p are indicated in each graph. Solid and dashed lines show the mean and 95% confidence intervals, respectively, following linear-regression analysis; the symbols show the r-Pearson, *p*-values, and regression formulas. The r-Pearson reflects the correlation, and the *p*-value indicates the significance of the correlation.

Next, we investigated the correlation between SPX and other parameters. We found a mild negative correlation between glucose (Figure 3C; SPX vs. glucose: r = −0.4843, *p* < 0.01), TG (Figure 3D; SPX vs. TG: r = −0.3472, *p* < 0.0516), and SPX. However, in the second case (TG vs. SPX correlation), we observed only a statistical trend. A positive correlation was found between glucagon concentration and SPX (Figure 3B; SPX vs. glucagon: r = 0.341, *p* < 0.05) and NEFA concentration and SPX (Figure 3E; SPX vs. NEFA: r = 0.4377, *p* < 0.05). We did not find the correlation between insulin and SPX concentration.

### 3.5. Effect of Fasting on SPX1, GALR2, and GALR3 mRNA Expression in Breast Muscle, Liver, and Fat Tissue

**Figure 4:**
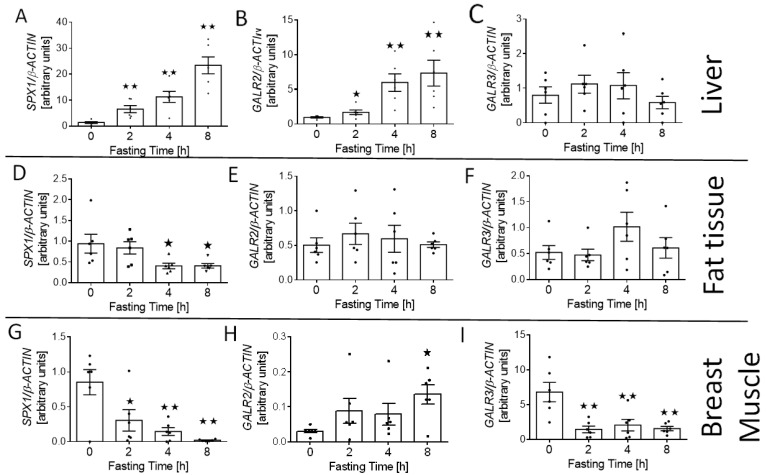
*SPX1*, *GALR2*, and *GALR3* mRNA changes in liver (**A**–**C**), fat tissue (**D**–**F**), and muscle (**G**–**I**) in response to short-term food deprivation. Results are shown as the means ± SEM for each value (*n* = 6). The dots represent the individual values obtained. Statistically significant changes are marked as asterisk symbol * *p* < 0.05 and ** *p* < 0.01 compared to time 0.

We also investigated the effect of fasting on the expression of SPX (*SPX1*) and its receptor (*GALR2* and *GALR3*) mRNA in the tissues involved in glucose and lipid homeostasis—liver, fat tissue (visceral fat), and muscle. We found that starving increased the relative level of *SPX1* (Figure 4A; *p* < 0.01) and *GALR2* mRNA (Figure 4B; 2 h *p* < 0.05; 4 and 8 h *p* < 0.01) in the liver, whereas we did not observe any differences in the expression of the *GALR3* gene in this digestive gland (Figure 4C). We noted that fasting decreased *SPX1* expression in fat tissue and muscle (Figure 4D; 4 and 8 h *p* < 0.05; and Figure 4G; 2 h *p* < 0.05, 4 and 8 h *p* < 0.01). We also observed that after 8 h of fasting, the expression of *GALR2* increased in the muscle (Figure 4H; *p* < 0.05), whereas the level of *GALR3* mRNA was reduced after 2, 4, and 8 h of food deprivation (Figure 4I; *p* < 0.01). At the same time, no changes in the level of *GALR2* and *GALR3* mRNA in the adipose tissue were observed (Figure 4E,F).

## 4. Discussion

In the present study, we showed for the first time that *SPX*, *GALR2*, and *GALR3* are widely expressed in the tissues of broiler chickens. We also found that a fasting state increased the SPX concentration in the serum blood, which was correlated with other metabolic markers, including glucose and glucagon levels. Moreover, we proved that the expression of *SPX1*, *GALR2*, and *GALR3* is regulated by food deprivation in the tissues involved in glucose homeostasis.

Currently, nothing is known about the expression of the *SPX* gene in birds. However, the data obtained from experiments carried out on mammals and fish, as well as genomic analyses, indicate that this peptide is widely distributed in many animal species. Research performed by Porzionato et al. in 2010 showed that SPX is expressed in many tissues in rats. The mRNA of SPX was observed in all examined tissues [18]. Similar results were also obtained using *Schizothorax prenanti* as a model for studies in fish [7]. Therefore, in the first step of our experiment, we investigated the level of *SPX1* mRNA expression in various tissues of chickens. We observed the mRNA expression of this peptide in all tested tissues, which suggests that SPX is a peptide with very broad action. We also confirmed the previous results regarding the expression of the chicken galanin receptors *GALR2* [19], which are also the receptors for the SPX, whereas studies performed by Boschiero et al. showed that *GALR3* is also expressed in broiler chickens. Moreover, these studies indicated that the *GALR3* gene is one of the of 260 genes that are important to metabolic pathways (such as cell signaling and interaction and cellular functions) and harbor variants with high-potential functional effects, which makes them very susceptible to various kinds of polymorphisms (e.g., single-nucleotide polymorphism (SNPs), frameshifts, etc.) [20]. Moreover, the variable expression of SPX in different tissues may also be due to the different role of this peptide in the regulation of the metabolism of particular systems. Currently, in the literature, it has been shown that SPX plays a key role in processes such as immune processes [21], regulation of the liver [5] and pancreas functions [10], and gastrointestinal tract functions by effecting the rate of the passage of gastric contents [9] in mammals. Given the very wide distribution of SPX in both the central nervous system and peripheral tissues, it is difficult to indicate which are its main source. However, based on the literature data and the results obtained in this study, we can generally state that the main sources of this peptide are tissues involved in carbohydrate–lipid metabolism and related to the regulation of food intake and energy management, such as the gastrointestinal tract, liver, pancreas, muscles, and others [2,22,23]. Moreover, changes in the blood-serum concentration and expression of this peptide indicate the possibility of SPX acting via multiple signaling pathways, both through endocrine action and autocrine/paracrine interactions [23].

The increased expression of SPX in these tissues also observed in our research indicates that SPX could also be very important in the context of regulating the functions of these organs in broilers.

The restriction of food in living organisms leads to an increase in metabolic processes aimed at activating the energy reserves accumulated in the supply of carbohydrates (glycogen) and lipids stored mainly in adipose tissue in various parts of the body. To date, most of the published studies on the effects of SPX focused on SPX’s effects on lipid metabolism and pathological conditions [5,24]. Therefore, we assessed whether a metabolic imbalance, i.e., short-term fasting, is associated with changes in the concentration of SPX in the blood serum and whether the changes in its expression in tissues are related to carbohydrate–lipid metabolism. A breakthrough for research on SPX in the context of lipids and carbohydrates occurred in the work of Walewski et al., who showed in 2014 that the amount of circulating SPX in the blood serum decreases under obesity and that the gene encoding this protein is one of the most strongly down-regulated genes in adipose tissue under this pathological condition in humans and mice [6]. SPX is likewise important in the context of our work, as Walewski et al. also showed the presence of changes in SPX concentration and expression in obese animals with excess energy (dominance of a positive-energy balance). This is the opposite of our results. Our results showed an increase of the SPX concentration in blood serum after fasting, which seems to be consistent with the results obtained in mammalian models. However, surprisingly, the level of *SPX1* expression in broiler adipose tissue and muscle decreased. At the same time, we observed up-regulation of *SPX1* mRNA expression in the liver. In our opinion, the trend of changes in gene expression in different tissues can be explained in two ways. The first is the relatively short time of fasting, which activates the energy reserves stored in the form of liver glycogen. However, the opposite tendency in muscle tissue is difficult to explain in this context. On the other hand, the metabolic specificity of chicken broilers causes this metabolism to be directed, to a greater extent, to the muscles, energy use, and growth of this tissue in these birds [25]. The second explanation might be the activation of energy reserves by the secretion of SPX into the blood and its subsequent effect on target tissues, which explains the increased concentration of this peptide in the blood.

The starvation process is also associated with the stimulation of metabolic processes related to the release of stored energy substances, e.g., in the form of nonesterified fatty acids (NEFA), which result from the intensification of lipolytic processes as well as activation of genes involved in lipid metabolism in fat tissue and liver [26,27]. PDK4 is considered to be involved in re-esterification of fatty acid derived from lipolysis during fasting. [26,28]. Previous studies performed on mammals showed that SPX is able to regulate both of these processes. It was shown that SPX regulates lipolysis by influencing the expression of the hormone-sensitive lipase HSL and by lipogenesis and adipocytes differentiation decreasing *Pparγ* and fatty-acid-synthase (FAS) expression in humans and rodents [4,24]. We also demonstrated that the SPX and NEFA concentrations in blood serum increased after food deprivation, which may indirectly indicate that the increase in NEFA level could be caused by the action of secreted SPX.

In summary, in this study, we showed, for the first time, the tissue mRNA expression of SPX broiler chicken. We also demonstrated the presence of changes in the concentration of this peptide in blood in response to short-term fasting. Furthermore, we showed that food deprivation leads to changes in the expression of the SPX/GALRs system in tissues related to glucose metabolism, such as liver, fat tissue, and muscle. Moreover, the concentration of this peptide is correlated with other metabolic and hormonal indicators of food deficiencies. The obtained results indicate that SPX may be a potential regulator of metabolism in broiler chicken and might be the starting point for further studies on the role of SPX in the metabolism of broiler chicken.

## Figures and Tables

**Table 1:** Composition and nutrient contents of the diets.

Ingredients (g/100 g, As-Fed Basis)	Starter	Grower
Maize	51.81	54.7
Soybean meal	38.1	34.7
Soybean oil	5.58	6.57
Monocalcium phosphate	1.88	1.66
Premix ^1^	1.00	1.00
Limestone (<2 mm)	0.38	0.29
NaHCO_3_	0.33	0.30
NaCl	0.22	0.26
HCl-Lysine	0.31	0.19
DL-Methionine	0.22	0.14
L-Threonine	0.13	0.05
L-Valine	0.05	0.14
**Calculated Nutrient Composition (g/100 g Unless Otherwise Stated)**		
AME_N_ [MJ/kg]	12.8	13.2
Crude protein	23.0	21.5
Ca	0.96	0.87
Available P	0.48	0.43
Available Lys	1.28	1.15
**Determined (g/100 g)**		
Crude protein	23.4	21.1
Crude fat	6.36	7.72
Ca	0.88	0.80
Total P	0.61	0.59

^1^ provides per kg diet: IU: vit. A—11,250, cholecalciferol—2500; mg: vit. E 80, menadione 2.50, vit. B12 0.02, folic acid 1.17, choline 379, D-pantothenic acid 12.5, riboflavin 7.0, niacin 41.67, thiamin 2.17, D-biotin 0.18, pyridoxine 4.0, ethoxyquin 0.09, Mn 73, Zn 55, Fe 45, Cu 20, I 0.62, Se 0.3, and salinomycin 60.

**Table 2:** Primer sequence.

Primer	Sequence 5′ > 3′	Product Size (bp)	Accession No.	Efficiency
*SPX1* (Forward)	GTGCACAGGGACGTCGATT	95	KT235743.1	98.78%
*SPX1* (Reverse)	AGGATTTGTGTTTTGGCTGCG			
*GALR3* (Forward)	GCTGGAATGCCTCCTCTGAC	373	NM_001130585.1	97.67%
*GALR3* (Reverse)	CGGGACTTCAGTGGATAGCG			
*GALR2* (Forward)	ATCCCGAGTCGGTGCTCAT	583	NM_001128063	98.11%
*GALR2* (Reverse)	CTCCAAAGGTAGCGAATGGT			
*β-ACTIN* (Forward)	CCCAGACATCAGGGTGTGATG	123	NM_205518	102.86%
*β-ACTIN* (Reverse)	GTTGGTGACAATACCGTGTTCAAT			

**Table 3:** Effect of fasting on the metabolic and hormonal profiles in broiler chickens. Results are shown as the means ± SEM for each value (*n* = 8). Statistically significant changes are marked as * *p* < 0.05 and ** *p* < 0.01 compared to time 0.

Variable	Fasting Time			
	0 h	2 h	4 h	8 h
	[mg/dL]			
Glucose	186.9 ± 3.37	168.5 ± 9.25	150.6 ± 5.18 **	152.8 ± 9.41 **
TG	119.8 ± 5.58	97.96 ± 6.53	94.9 ± 7.39 *	91.5 ± 6.23 *
Cholesterol	125.6 ± 3.66	127.6 ± 5.83	131.2 ± 4.12	126.5 ± 2.59
	[mmol/L]			
NEFA	0.438 ± 0.016	0.535 ± 0.03	0.575 ± 0.044 *	0.631 ± 0.047 **
	[g/dL]			
Albumin	1.473 ± 0.038	1.414 ± 0.05	1.462 ± 0.036	1.453 ± 0.018
Total protein	3.116 ± 0.110	3.062 ± 0.061	2.997 ± 0.067	3.018 ± 0.073
	[ng/mL]			
Insulin	0.444 ± 0.073	0.349 ± 0.071	0.427 ± 0.071	0.122 ± 0.037 *
	[pg/mL]			
Glucagon	378.9 ± 19.99	474.6 ± 37.80	537.6 ± 52.53 *	663.2 ± 20.97 **

TG—triglicerides; NEFA—Non-esterified fatty acids

## Data Availability

The data presented in this study are available on reasonable request from the corresponding author.

## Abbreviations

*SPX1*—spexin gene; SPX—spexin protein; *GALR2*—galanin receptor 2 gene, GALR2—galanin receptor 2 protein; *GALR3*—galanin receptor 3 gene, GALR3—galanin receptor 3 protein; SPX/GALRs—spexin–galanin receptors system; RT-qPCR—reverse transcription qPCR; TG—triglicerides; TChol—total cholesterol, NEFA—nonesterified fatty acids; SEM—standard error of the mean; DM—dry matter; CP—crude protein; CF—crude fat, SNP—single-nucleotide polymorphism.
